# A new species of diplectanid (Monogenoidea) from *Paranthias colonus* (Perciformes, Serranidae) off Peru

**DOI:** 10.1051/parasite/2015011

**Published:** 2015-03-09

**Authors:** Marcelo Knoff, Simone Chinicz Cohen, Melissa Querido Cárdenas, Jorge M. Cárdenas-Callirgos, Delir Corrêa Gomes

**Affiliations:** 1 Laboratório de Helmintos Parasitos de Vertebrados, Instituto Oswaldo Cruz, FIOCRUZ Av. Brasil 4365 Rio de Janeiro, RJ Brazil; 2 Laboratório de Helmintos Parasitos de Peixes, Instituto Oswaldo Cruz, FIOCRUZ Av. Brasil 4365 Rio de Janeiro, RJ Brazil; 3 Museo de Historia Natural, Facultad de Ciencias Biológicas, Universidad Ricardo Palma (URP) Av. Benavides 54440 Lima 33 Peru

**Keywords:** *Pseudorhabdosynochus jeanloui* n. sp., Monogenea, Diplectanidae, *Paranthias colonus*, Fish

## Abstract

*Pseudorhabdosynochus jeanloui* n. sp. (Monogenoidea, Diplectanidae) is described from specimens collected from the gills of the Pacific creolefish, *Paranthias colonus* (Perciformes, Serranidae) from a fish market in Chorrillos, Lima, Peru. The new species is differentiated from other members of the genus by the structure of its sclerotized vagina, which has two spherical chambers of similar diameter. This is the first *Pseudorhabdosynochus* species described from the Pacific coast of America, the third species of the genus reported from South America and the first described from a member of *Paranthias*.

## Abstract (fr)

*Pseudorhabdosynochus jeanloui* n. sp. (Monogenoidea, Diplectanidae) est décrit de spécimens collectés sur les branchies de la badèche du Pacifique, *Paranthias colonus* (Perciformes, Serranidae) d’un marché aux poissons de Chorrillos à Lima au Pérou. La nouvelle espèce se différencie des autres membres du genre par la structure de son vagin sclérifié, qui a deux chambres sphériques de diamètre similaire. Ceci est la première espèce de *Pseudorhabdosynochus* décrits de la côte Pacifique de l’Amérique, la troisième espèce du genre rapportée d’Amérique du Sud, et la première décrite d’une espèce de *Paranthias*.

## Introduction

*Pseudorhabdosynochus* Yamaguti, 1958 has a very complex nomenclatural history, which was elucidated by Kritsky & Beverley Burton [14] and further confirmed by Justine [8]. Domingues & Boeger [2], in a revision of the family, enlisted 52 valid species from *Pseudorhabdosynochus*. After this date, 25 more species were described. The species of *Pseudorhabdosynochus* mostly parasitize fish belonging to *Epinephelus* and have been described from different regions of the world [1, 2, 6–12, 17, 19–23].

Infections of *Pseudorhabdosynochus* species in captive fish populations can cause substantial economic losses to the fishing industry [24]. The accurate identification of members of *Pseudorhabdosynochus* to species is central in studying transmission patterns and is important for control of the diseases they cause, particularly given that there are currently no effective treatment strategies for these diseases [15].

During examination on fish from a market in Chorrillos, Lima, Peru, a member of *Pseudorhabdosynochus* was found parasitizing *Paranthias colonus* (Valenciennes, 1846) (Perciformes, Serranidae), which represents a new species for the genus.

## Material and methods

Two specimens of *Paranthias colonus* (total length 31–35 cm, weight 450–500 g) were obtained from a market in Chorrillos, Lima, Peru in November 2010. The gills were removed and placed in vials containing 1:4000 formalin solution. After 1 h, the vials were vigorously shaken and fixed in ethanol 70° GL. In the laboratory, the parasites were collected with the aid of a stereoscopic microscope and stored. Some specimens were mounted unstained in Hoyer’s medium [3] for study of the sclerotized parts and others were stained with Langeron’s carmine [13], cleared in beechwood creosote and mounted in Canada balsam as permanent slides. All specimens were observed and photographed in a Zeiss Axioskop brightfield microscope, equipped with a millimeter ocular, differential interference contrast (DIC) optics, and a Sony MPEGEX digital camera. The specimens were drawn using an Olympus BX 41 brightfield microscope equipped with a drawing tube. All measurements are in micrometers; the mean is followed by the range in parentheses and the number of specimens measured when more than two. Type-specimens are deposited in the Helminthological Collection of Instituto Oswaldo Cruz (CHIOC), Rio de Janeiro, Brazil and Muséum National d’Histoire Naturelle, Paris, France (MNHN).

Specimens used for scanning electron microscopy (SEM) were fixed in 70% alcohol, washed in 0.1 M cacodylate buffer pH 7.2 and postfixed in a solution containing 1% osmium tetroxide and 0.8% potassium ferricyanide pH 7.2 for 1 h. Subsequently, they were dehydrated through a graded ethanol series, dried by means of the critical point method with CO_2_ and sputter-coated with gold; they were examined in the Plataforma de Microscopia Eletrônica Rudolf Barth (IOC-Fiocruz) using a JEOL JSM-6390 LV scanning electron microscope at an accelerating voltage of 30 kV.

## *Pseudorhabdosynochus jeanloui* n. sp.

urn:lsid:zoobank.org:act:9A3F6D01-C69F-4537-8843-D2311EB048A4

Type-host: *Paranthias colonus* (Valenciennes, 1846) (Perciformes, Serranidae).

Type-locality: off Chorrillos, Lima, Peru.

Specimens studied: Holotype, CHIOC 38.016a; 8 paratypes, CHIOC 38.016 b-i; 2 paratypes, MNHN HEL 540-541.

Etymology: The new species is named after Dr. Jean-Lou Justine in recognition of his contribution to knowledge of Monogenoidea, especially to species of *Pseudorhabdosynochus*.

### Description (Figs. 1–3)

Body 781 (600–930; *n* = 7) long by 213 (105–300; *n* = 7) wide. Tegument scaly, posterior region with scales on ventral and dorsal surfaces from squamodiscs to level of anterior part of testis. Anterior region with three pairs of lateral head organs; two groups of cephalic glands lateral to pharynx, both with numerous ducts leading to head organs. Two pairs of eye spots; anterior pair slightly smaller than posterior one. Pharynx spherical 51 (43–55; *n* = 5) × 50 (43–55; *n* = 5). Esophagus very short, intestinal bifurcation immediately follows pharynx.

**Figure 1.**
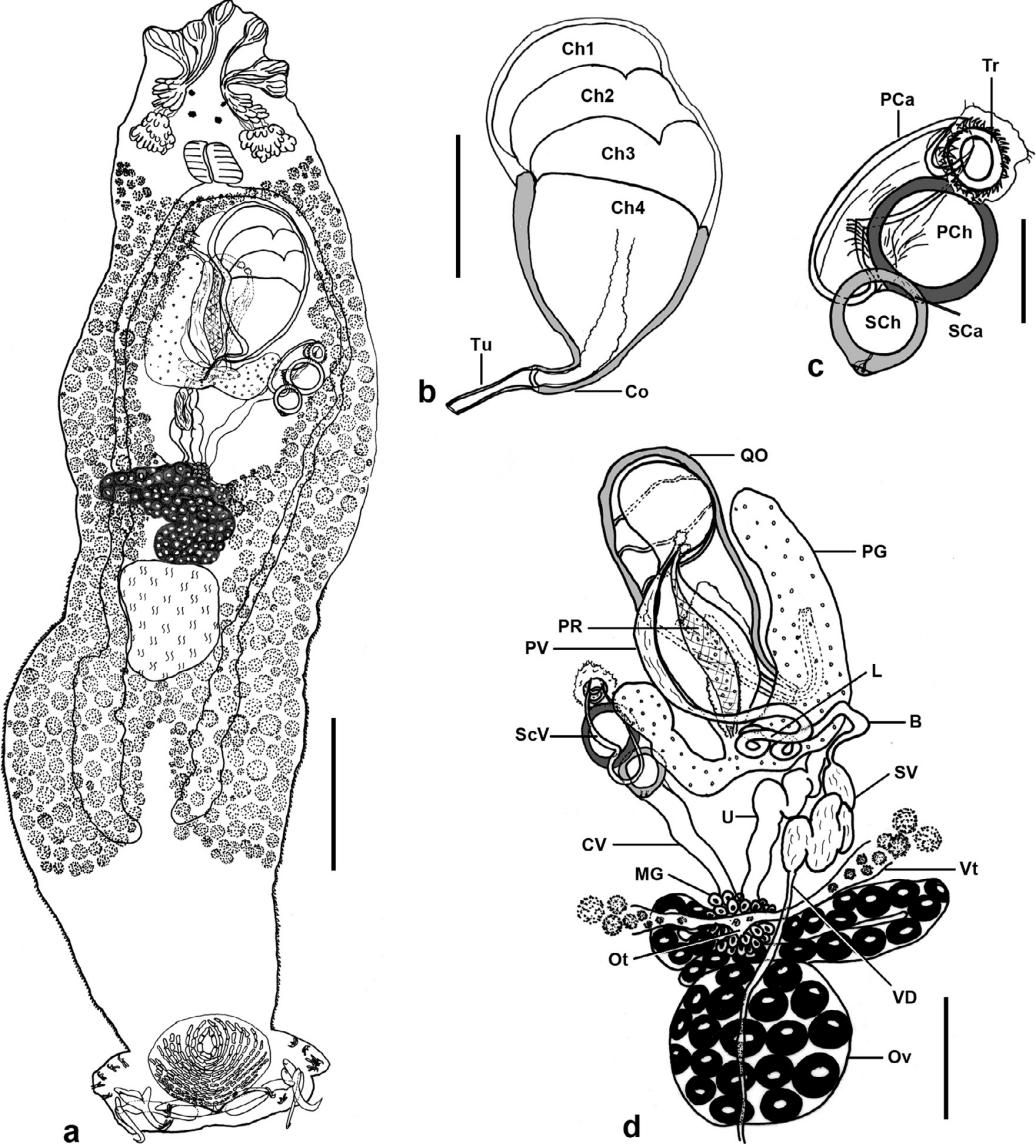
*Pseudorhabdosynochus jeanloui* n. sp. from *Paranthias colonus*. **a**, composite, mainly from holotype, Hoyer, tegumental scales only on edges, ventral view. **b**, quadriloculate organ, holotype, Hoyer, ventral view. Ch1-4, chambers; Co, cone; Tu, tube. **c**, sclerotized vagina, ventral view. Tr, trumpet; PCa, primary canal; PCh, primary chamber; SCa, secondary canal; SCh, secondary chamber. **d**, male and female organs, testis not represented, paratype, carmine, dorsal view. VD, vas deferens; SV, seminal vesicle; B, bend; L, loops; PV, deferent enlarged posterior vesicle; PR, prostatic reservoir; PG, prostatic gland; QO, quadriloculate organ; Ov, ovary; Ot, ootype; Vt, vitelline ducts; MG, Mehlis’s glands; U, uterus; CV, unsclerotized canal from secondary chamber of vagina to ootype; ScV, sclerotized vagina. Scale bars: a, 100 μm; b, d, 40 μm; c, 20 μm.

**Figure 2.**
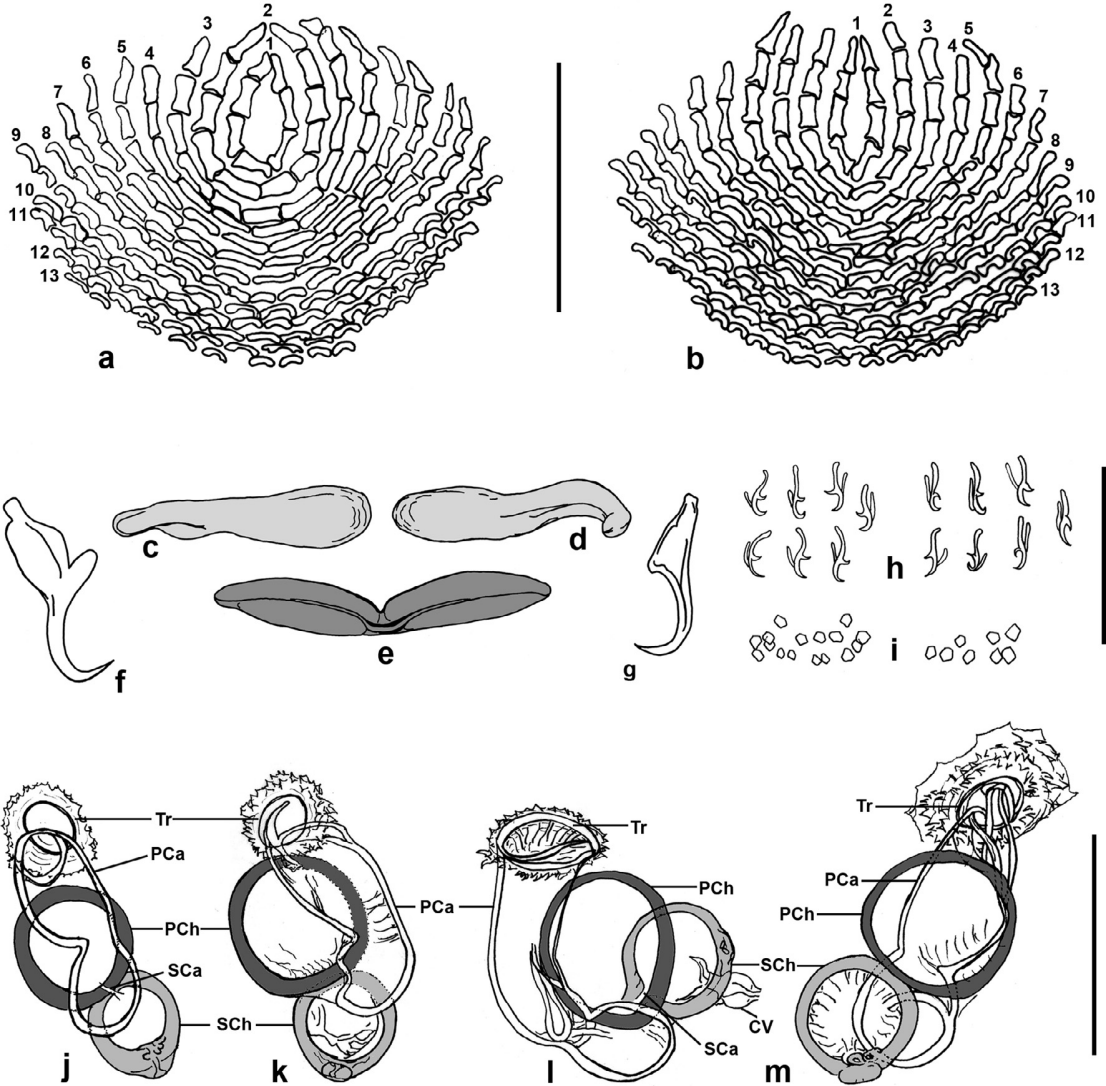
*Pseudorhabdosynochus jeanloui* n. sp. from *Paranthias colonus*. **a, b**, squamodiscs; **a**, ventral, and **b**, dorsal, from holotype, Hoyer. **c, d**, dorsal bars, holotype, Hoyer, ventral view. **e**, ventral bar, holotype, Hoyer, ventral view. **f–g**, hamuli; **f**, ventral hamulus, and **g**, dorsal hamulus, paratype, Hoyer, ventral view. **h**, hooklets from various specimens, ventral view. **i**, tegumental scales, holotype, Hoyer. **j–m**, sclerotized vaginae of various specimens; Tr, trumpet; PCa, primary canal; PCh, primary chamber; SCa, secondary canal; SCh, secondary chamber; CV, unsclerotized canal from secondary chamber to ootype. j, paratype, carmine; k–m, paratypes, Hoyer; j–l, dorsal view; m, ventral view. Scale bars: 40 μm.

**Figure 3.**
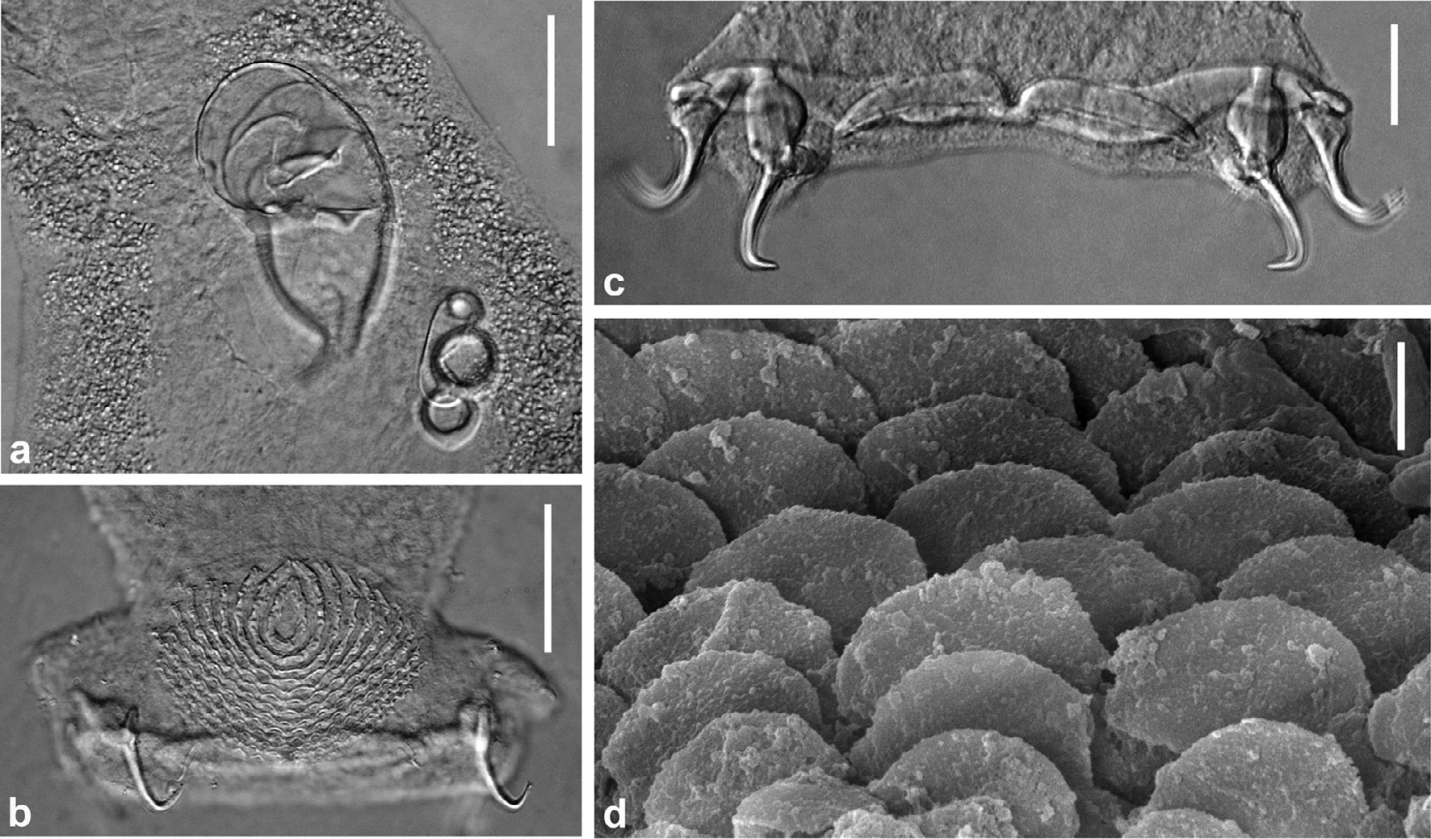
*Pseudorhabdosynochus jeanloui* n. sp. from *Paranthias colonus*. **a–c**, brightfield differential interference contrast microscope images. **a**, detail of quadriloculate organ and sclerotized vagina, holotype, Hoyer, ventral view. **b**, haptor, detail of ventral squamodisc and ventral hamuli, holotype, Hoyer, ventral view. **c**, haptor, detail of ventral and dorsal hamuli and bars, paratype, Hoyer, ventral view. **d**, scanning electron microscope image, detail of rodlet rows of a squamodisc, ventral view. Scale bars: a–b, 40 μm; c, 20 μm; d, 2 μm.

Haptor differentiated from the rest of the body, expanded laterally, width 158 (135–185; *n* = 9), provided with two similar squamodiscs, two pairs of anchors and 14 hooks. Squamodiscs made up of rows of rodlets; central rows form closed rings. Ventral squamodisc, length 50 (41–60; *n* = 5), width 76 (46–120; *n* = 5), with 10–13 (*n* = 4) rows of rodlets and 1–2 closed rings. Dorsal squamodisc, length 52 (43–58; *n* = 5), width 72 (55–80; *n* = 5), with 10–13 (*n* = 5) rows of rodlets and 1 closed ring. Ventral anchor with well-differentiated superficial and deep roots, bent shaft and point, outer length 42 (37–45; *n* = 6), inner length 31 (29–33; *n* = 5). Dorsal anchor with indistinct roots, straight shaft and point, outer length 36 (34–39; *n* = 6), inner length 22–24 (*n* = 3). Dorsal (lateral) bars straight, with flattened medial extremity and thick cylindrical lateral extremity, length 55 (46–65; *n* = 18), width 14 (12–17; *n* = 13). Ventral bar with median constricted portion and tapered ends, length 74 (66–92; *n* = 11), width 11 (10–14; *n* = 10).

Testes sub-spherical, intercaecal. Vas deferens emerges from antero-sinistral part of testis, enlarges forming seminal vesicle (located in middle region of body), forms bent, transforms in duct which loops, then directs forward, enlarges distally as a deferent posterior vesicle, then connects with quadriloculate male copulatory organ. Prostatic reservoir conspicuous, with visible transverse muscular fibers, connects with quadriloculate organ. Prostatic glands medio-dextral, converge at the base of prostatic reservoir.

Quadriloculate organ with four chambers, inner length 89 (73–105; *n* = 8). Fourth chamber more sclerotized than others, opens into short sclerotized cone 9 (5–10; *n* = 9), prolonged by sclerotized tube 17 (15–20; *n* = 7); end of tube prolonged by unsclerotized filament of variable length.

Ovary subequatorial intercaecal, pre-testicular, encircles right intestinal caecum; oviduct passes medially to form ootype, which is surrounded by Mehlis’ gland, short, open into uterus. Uterus dextral. Unsclerotized vagina inconspicuous.

Sclerotized vagina with conspicuous trumpet, followed by primary canal and two spherical chambers located along canal. Trumpet wide, diameter larger than that of primary canal. Primary canal with thin wall and constriction at mid-length. Primary chamber located at posterior extremity of primary canal, spherical, with regular thin wall. Secondary canal (communication between primary chamber and secondary chamber) very short, reduced to thin perforation in width of wall of two adjacent chambers. Secondary chamber spherical, with regular thin wall, smaller than primary chamber and generally posterior to it. Unsclerotized canal from sclerotized vagina to ootype connected to secondary chamber. Total length of sclerotized vagina 55 (47–63; *n* = 10), primary chamber external diameter 22 (20–25; *n* = 10), secondary chamber external diameter 18 (15–21; *n* = 10). Only two eggs observed, length 52–62, width 42.

By scanning electron microscopy, rodlets of squamodiscs can be observed as semi-circular scales.

## Discussion

*Pseudorhabdosynochus* is unique among Diplectanidae by having a quadriloculate male copulatory organ. The genus is also characterized by a sclerotized vagina, which is the key structure for identifying species of *Pseudorhabdosynochus*. By these characteristics, the new species is easily accommodated to the genus [18].

*Pseudorhabdosynochus jeanloui* n. sp. can be distinguished from others in the genus by the morphology of the sclerotized vagina, composed of a robust elongate trumpet and two spherical chambers, with the primary chamber larger than the secondary. As in *P. jeanloui* n. sp., several species of the genus present a vagina with two chambers and the secondary chamber smaller than the primary, including *P. epinepheli* (Yamaguti, 1938), *P. minutus* Justine, 2007, *P. morrhua* Justine, 2008, *P. malabaricus* Justine & Sigura, 2007, and *P. auitoe* Justine, 2007. *Pseudorhabdosynochus morrhua* Justine, 2008 has a secondary chamber larger than the primary, while the chambers are of similar size in *P. duitoe* Justine, 2007, *P. dionysos* Schoelinck & Justine, 2011 and *P. bacchus* Sigura, Chauvet & Justine, 2007. *Pseudorhabdosynochus jeanloui* n. sp. differs from all of these species by the measurements of the total length and by the proportion of the chambers, which are significantly larger [5, 7, 11, 20, 22, 25].

The other similar species is *P. venus* Hinsinger & Justine, 2006, which also has a secondary chamber smaller than the primary chamber, but the chambers are smaller than in *P. jeanloui* n. sp.: primary chamber 20–25 in *P. jeanloui* vs. 12–16 (carmine) and 16–22 (picrate) in *P. venus*, secondary chamber 15–21 in *P. jeanloui* vs. 11–13 (carmine) and 12–17 (picrate) in *P. venus* [4]. Besides this, *P. jeanloui* n. sp. has a similar quadriloculate organ, considering that the fourth chamber is more sclerotized than the other three.

A species of *Pseudorhabdosynochus* has recently been reported [16] from the same host off Peru, but not described, and probably represents the same species as the one described here.
